# Effects of additional anterior body mass on gait

**DOI:** 10.1186/s12884-016-0893-0

**Published:** 2016-05-16

**Authors:** Simone V. Gill, Maureen Ogamba, Cara L. Lewis

**Affiliations:** Department of Occupational Therapy, Boston University, 635 Commonwealth Avenue, Boston, MA 02215 USA; Program in Rehabilitation Sciences (PhD), Boston University, 635 Commonwealth Avenue, Boston, MA 02215 USA; Department of Medicine, Boston University, 715 Albany Street, Boston, MA 02218 USA; Department of Physical Therapy and Athletic Training, Boston University, 635 Commonwealth Avenue, Boston, MA 02215 USA

**Keywords:** Gait, Body mass, Walking, Velocity, Speed

## Abstract

**Background:**

Gradual increases in mass such as during pregnancy are associated with changes in gait at natural velocities. The purpose of this study was to examine how added mass at natural and imposed slow walking velocities would affect gait parameters.

**Methods:**

Eighteen adult females walked at two velocities (natural and 25 % slower than their natural pace) under four mass conditions (initial harness only (1 kg), 4.535 kg added anteriorly, 9.07 kg added anteriorly, and final harness only (1 kg)). We collected gait kinematics (100 Hz) using a motion capture system.

**Results:**

Added anterior mass decreased cycle time and stride length. Stride width decreased once the mass was removed (*p* < .01). Added mass resulted in smaller peak hip extension angles (*p* < .01). The imposed slow walking velocity increased cycle time, double limb support time and decreased stride length, peak hip extension angles, and peak plantarflexion angles (*p* < .01). With added anterior mass and an imposed slow walking velocity, participants decreased cycle time when mass was added and increased cycle time once the mass was removed (*p* < .01).

**Conclusions:**

Gait adaptations may be commensurate with the magnitude of additional mass when walking at imposed slow versus natural velocities. This study presents a method for understanding how increased mass and imposed speed might affect gait independent of other effects related to pregnancy. Examining how added body mass and speed influence gait is one step in better understanding how women adapt to walking under different conditions.

## Background

Gradual increases in mass are associated with corresponding changes in gait. For example, during pregnancy, women gain from 11–16 kg [1]. The mass of the trunk increases over the course of pregnancy with most of the additional mass positioned anteriorly [2, 3]. Pregnant women exhibit concurrent alterations in their walking parameters. For example, pregnant women decrease stride length, which results in taking shorter steps [4]. Over the course of pregnancy, changes in women’s gait continue to show a linear trend; women increase step width [5], decrease stride length [4–6], and decrease step length [4, 6]. However, a range of evidence exists with regard to the influence of pregnancy on women’s walking velocity. Some studies show decreases in walking velocity across weeks of pregnancy [7–9] while others reveal no differences in velocity [5, 10].

The gait modifications discussed above may increase stability for pregnant women and subsequently counteract poor balance [11]. However, these modifications are positively correlated with difficulty recovering balance once it has been lost due to decreased postural stability [4, 12], which leaves pregnant women susceptible to falls [12, 13]. Public health studies show that approximately 27 % of women report falls during pregnancy [13]. Falls during pregnancy can have serious consequences [14]. As a result of falling, pregnant women sustain injuries such as lower extremity fractures [15]. Even more alarming, when falls during pregnancy result in hospitalization, these falls are linked with a 4.4 fold increase in pre-term labor, a 2.1 fold increase in fetal distress, and a 2.9 fold increase in fetal hypoxia [15].

Despite the association between added body mass and gait on the risk of falls, our current knowledge about this relationship is limited to walking at natural (i.e., self-selected) paces. Research on changes in gait that occur during pregnancy documents the effect of a gradual increase in mass as women walk at self-selected paces. Some changes in biomechanics are seen in conjunction with reduced self-selected walking velocities. Previous literature also details the effects of added mass on gait stability [16, 17]. Therefore, we know little about whether walking at imposed slower velocities with added mass would result in altered lower limb sagittal plane hip, knee, and ankle kinematics and spatio-temporal gait patterns. The purpose of the current study was to examine how an addition of mass at both natural and imposed slow walking velocities would affect spatio-temporal and kinematic gait parameters. Fall risks are heightened for pregnant women and the effects of decreased walking velocities on gait with added mass are unclear. Therefore, we created a paradigm to safely test the effects of added anterior mass on gait with healthy, non-pregnant women. We hypothesized that simulating pregnancy with added anterior mass would alter women’s gait parameters. In particular, we were interested in examining the effect of an imposed slower velocity combined with an increase in mass on gait parameters.

## Methods

### Participants

Participants were recruited and tested at the Human Adaptation and Motor Development Laboratories at Boston University. Eighteen adult females participated (*M* age = 21.83 years, *SD* = 3.07). Table 1 includes participants’ demographics and anthropometrics. Participants had no known significant injuries affecting their gait or safe participation in the study such as foot deformities, orthopedic injuries, or cardiac, visual, hearing, and neuropathic conditions. The study was approved by the Boston University Institutional Review Board and conformed to the Declaration of Helsinki. Informed written and verbal consent were obtained from all participants before testing began. Written consent to publish indirect identifiers was obtained from participants (e.g., age).

**Table 1:** Demographic information for participants

Participant	Age	Height (m)	Mass (kg)
1	20	1.75	81.8
2	20	1.65	54.5
3	19	1.57	50
4	20	1.69	71.4
5	21	1.63	61.4
6	22	1.65	60.45
7	22	1.7	63.6
8	28	1.77	78.64
9	30	1.65	56.82
10	24	1.75	63.64
11	20	1.63	54.55
12	24	1.73	56.82
13	20	1.68	61.36
14	19	1.6	63.64
15	20	1.75	65.91
16	23	1.6	74.09
17	22	1.65	58.64
18	19	1.6	54.5
MEAN	21.83	1.67	62.88
SD	3.07	0.06	8.72

### Motion capture and treadmill

We collected lower extremity, pelvis and trunk kinematics (100 Hz) using a 10-camera Vicon motion capture system with a standard marker set of 29 markers and 4 quad markers placed bilaterally on the bony parts of the subject’s lower extremities and trunk [18]. Specifically, markers were placed bilaterally over the posterior aspect of the calcaneus, first and fifth metatarsal heads, medial and lateral malleoli, medial and lateral femoral epicondyle, greater trochanter, iliac crest, anterior superior iliac spine, and posterior superior iliac spine. To define the trunk segment, markers were placed bilaterally on the acromion processes, spinous process of 7th cervical vertebra, and xiphoid. Non-collinear marker clusters with 4 reflective markers were positioned bilaterally on the distal thigh and shank. The clusters were attached to body segments with neoprene wraps, velcro and pre-wrap. Commercial software (Visual3D, C-Motion, Inc, Rockville, MD) was used to extract gait parameters of interest. The spatio-temporal parameters were: mean cycle time, double limb support time, velocity, stride length, and stride width. The kinematic variables were: peak hip extension angle, peak hip flexion angle, peak knee flexion angle, peak knee extension angle, peak ankle plantarflexion angle, and peak ankle dorsiflexion angle. Motion capture data were collected as subjects walked on a split belt treadmill (Bertec Corporation, Columbus, OH).

### Artificial pregnancy belly

Participants wore a custom-built artificial pregnancy belly while walking. The artificial pregnancy belly consisted of a harness (1 kilogram (kg)) similar to a backpack with shoulder straps and a waist strap to which additional mass was added. The additional mass, in the form of ankle cuff weights, were loaded into the artificial belly and distributed horizontally in front of the abdomen, simulating advancing pregnancy. The mass of each cuff weight was 4.535 kg. The subjects were tested with two different sizes of the artificial pregnancy belly: 5–7 months (harness plus one cuff weight (5.535 kg total)) and 8–9 months (harness plus two cuff weights (10.07 kg total)). The loads were, on average, 9 % and 17 % of participants’ body weight for the 5.535 kg and 10.07 kg conditions respectively.

### Procedure

We determined participants’ natural walking velocity by instructing them to walk laps around the lab at their preferred pace with shoes. We timed them as they crossed a 5-m distance and used the average of 5 laps to determine their natural walking velocity. We used overground instead of treadmill walking to determine natural walking velocity because our pilot data in young, healthy adults showed no differences in natural walking velocity calculations using either method [19]. We then placed markers onto participants. They walked on the treadmill at their natural walking velocity under four conditions: initial harness only (1-kg (2.2-pounds:lbs) distributed around the trunk), harness with 4.535-kg (10-lbs) added anteriorly to the artificial pregnancy belly resulting in 5.535-kg total added mass, harness with 9.07-kg (20-lbs) added anteriorly to the artificial pregnancy belly resulting in 10.07-kg total added mass, and final harness only (1-kg). Participants then walked under the same four conditions at an imposed velocity 25 % slower than their natural walking pace. The reduction was based on pilot work indicating that, when instructed to walk at a slower pace than normal, the self-selected slow pace was approximately a 25 % reduction from the participant’s preferred pace.

#### Statistical Analyses

Using SPSS 20.0 statistical software, we conducted separate 2 velocity (natural, imposed slow) x 4 mass (initial harness only, 5.535-kg added mass, 10.07-kg added mass, final harness only) repeated measures (RM) analyses of variance (ANOVA) to examine differences in participants’ average cycle times, double limb support times, stride lengths, stride widths, peak hip extension angle, peak hip flexion angle, peak knee flexion angle, peak knee extension angle, peak ankle plantarflexion angle, and peak ankle dorsiflexion angle. For all tests, statistical significance was set at 0.05 (two-tailed). Post hoc analyses consisted of pairwise comparisons. We used Bonferroni corrections to prevent experiment-wise errors. Effect sizes for follow up pairwise comparisons are represented with Cohen’s *d* after each p-value [20]. Effect sizes can be interpreted as small, medium, or large based on absolute values of Cohen’s *d* (i.e., Cohen’s *d* may be a negative value, but interpreting the effect size is based on the absolute value): absolute values of Cohen’s *d* ≥ 0.2 = small effects, ≥ 0.5 = medium effects, and ≥ 0.8 = large effects.

## Results

### Effects of Added Mass

#### Spatio-temporal data

**Fig. 1:**
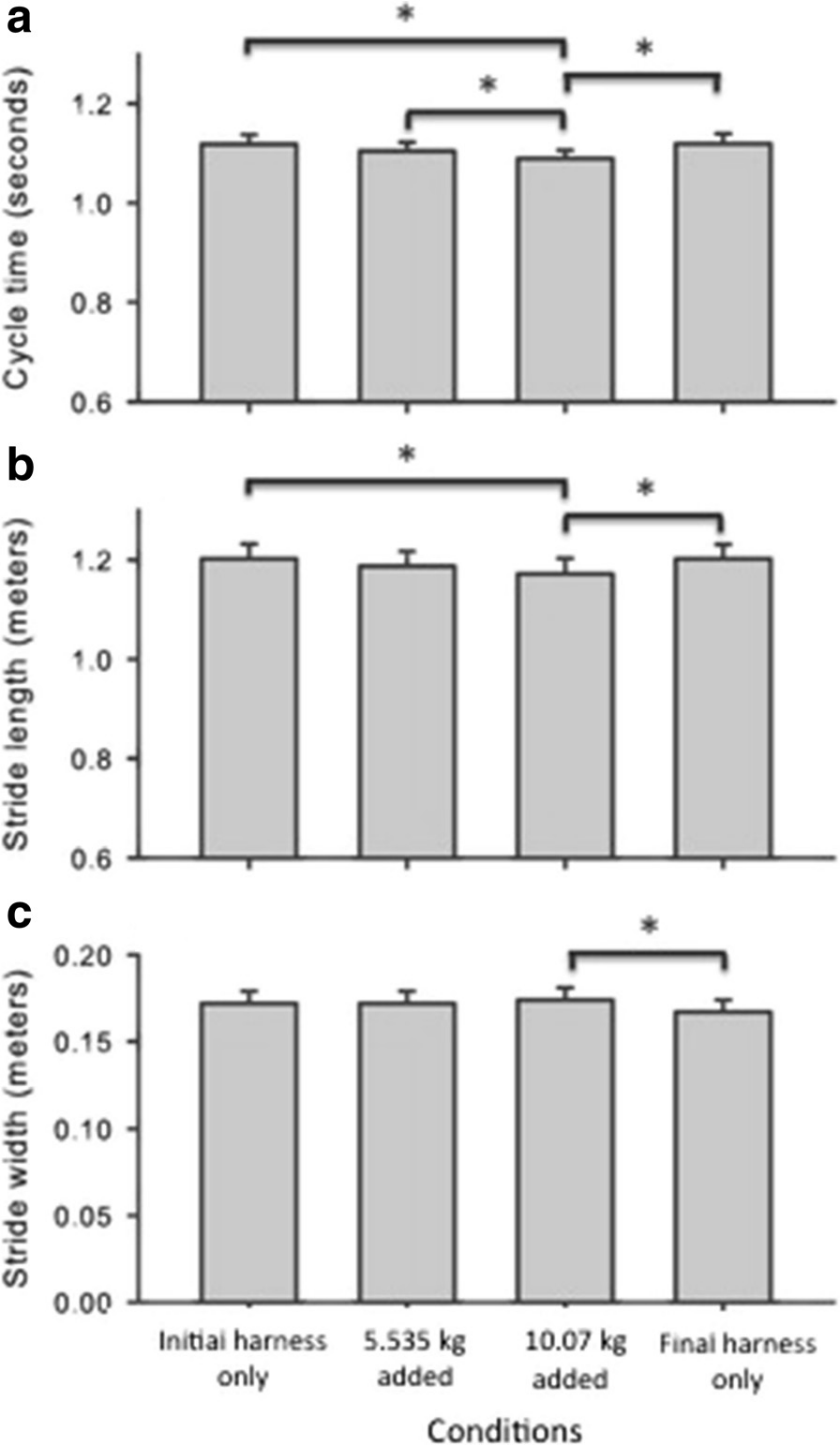
Spatio-temporal gait parameters by mass condition. Average cycle times (1**a**), stride lengths (1**b**), and stride widths (1**c**) are graphed for the initial harness only, 5.535 kg mass, 10.07 kg mass, and final harness only conditions. Bars represents standard errors. * = *p* < .01

Adding anterior mass influenced participants’ walking patterns. The results for mean cycle time revealed a main effect for condition (*F*(3,51) = 28.69, *p* < .001). Mean cycle times were lowest when participants wore the harness with added mass than when they had no mass added to the harness (*p* < .01; *d* from 0.50 to 1.50). In the 10.07-kg added mass condition, participants had the lowest mean cycle time compared to all other conditions (*p* < .001; *d* from 1.00 to 1.50, Fig. 1a). Analyses of stride length showed effects for condition (*F*(3,51) = 36.75, *p* < .001). Stride length was shorter during the 10.07-kg condition compared to both harness only conditions (*p* < .001; *d* from −0.50 to −1.60, Fig. 1b). Results for stride width were significant for condition (*F*(3,51) = 8.08, *p* < .01); participants decreased stride width during the final harness only condition once the anterior mass was removed (*p* < .01, *d* = 0.71, Fig. 1c).

Lower limb sagittal plane hip, knee, and ankle kinematic data. Analyses of the peak hip, knee and ankle sagittal plane angles showed a main effect for condition for each kinematic dependent variable (*p* < .05). Subjects had smaller peak hip extension angles during both added mass conditions compared to both harness only conditions (*p* < .01, *d* from −0.90 to -.1.44). They also had less peak hip extension when wearing the 10.07 kg versus 5.535 kg added mass (*p* < .01). Peak hip flexion angle was larger during the 10.07 kg added mass condition compared to both harness only conditions (*p* < .01, *d* 0.89 to 1.31). Peak knee flexion angle was larger when subjects wore the 10.07 kg versus 5.535 kg added mass (*p* < .01, *d* = 0.20). Peak knee extension angle was smaller at both anterior added mass conditions compared to the initial harness only condition (*p* < .01, *d* from 0.24 to 0.42). Subjects also had smaller peak knee extension when wearing 10.07 kg added mass compared to the final harness only condition (*p* < .01, *d* = −0.48). Peak ankle plantarflexion angle was smaller during the 10.07 kg added mass condition compared to the final harness only condition (*p* < .01, *d* = 0.96). While results for peak ankle dorsiflexion angle showed a main effect for condition (*F*(3,51) = 3.85, *p* < .05), follow up comparisons did not meet criteria for significance after the Bonferroni correction.

### Effects of Velocity

#### Spatio-temporal data

**Fig. 2:**
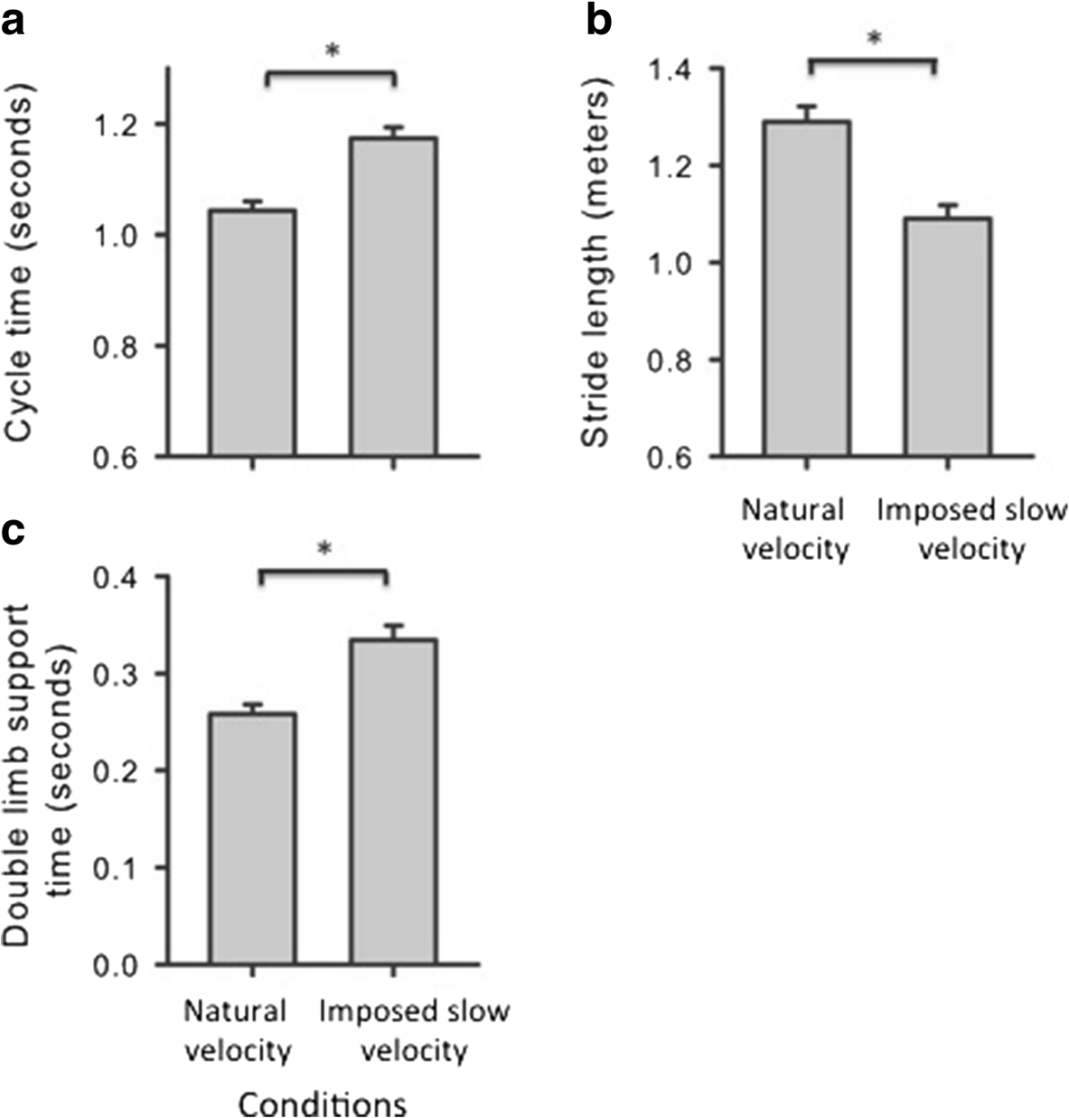
Spatio-temporal gait parameters by walking velocity. Average cycle times (2**a**), stride lengths (2**b**), and double limb support times (2**c**) are graphed for natural and imposed slow velocities. Bars represents standard errors. * = *p* < .01

The average natural walking velocity was 1.24 meters per second (m/s) (*Standard Deviation* (*SD)* = 0.17), and the average imposed slow walking velocity was 0.93 m/s (*SD* = 0.13). Participants’ spatio-temporal gait parameters were affected by walking at natural versus imposed slow velocities. We found a main effect for velocity on mean cycle time (*F*(1,17) = 788.32, *p* < .0001). At the imposed slow velocity, participants had higher mean cycle times than at the natural velocity (*p* < .001, *d* = −6.50, Fig. 2a). Analyses of stride length showed effects for velocity (*F*(1,17) = 1547.27, *p* < .0001). Stride length was shorter at the imposed slow versus the natural walking velocity (*p* < .001, *d* = 10.00, Fig. 2b). The results for double limb support time also showed a main effect for velocity (*F*(1,17) = 97.91, *p* < .001). Double limb support time was higher during the imposed slow versus the natural walking conditions (*p* < .001, *d* = −5.43, Fig. 2c). No differences were found for stride width for the effect of walking velocity (*p* > .05).

**Fig. 3:**
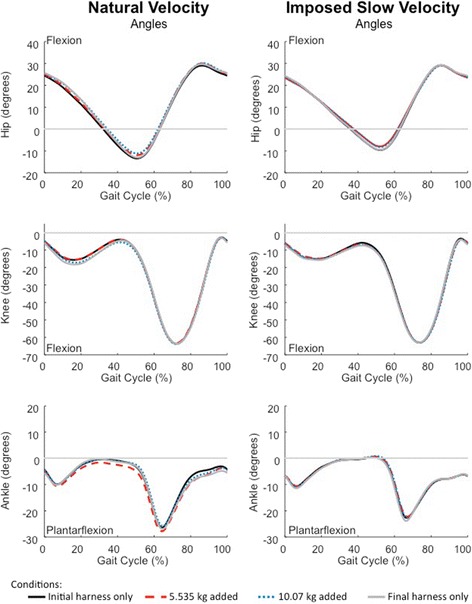
Hip, knee, and ankle kinematic data by walking velocity. Data are the mean of all subjects walking in the initial harness only (solid black line), 5.535 kg mass (large dashed line), 10.07 kg mass (small dashed line), and final harness only (solid gray line) conditions at the natural (left) and slow (right) velocities. Hip flexion, knee extension, and ankle dorsiflexion are all positive

Lower limb sagittal plane hip, knee, and ankle kinematic data. At the imposed slow velocity, subjects had smaller peak hip extension angles than at the natural velocity (*F*(1,17) = 18.38, *p* < .001, *d* = −2.17). They also had smaller peak hip flexion angles at the imposed slow velocity (*F*(1,17) = 22.24, *p <* .001, *d* = 2.78). No significant velocity effects were found for peak knee flexion or knee extension angles (*p* > .05). Peak ankle plantarflexion angle was smaller during the imposed slow versus the natural velocity (*F*(1,17) = 47.33, *p* < .001, *d* = −2.63). Peak ankle dorsiflexion angle was larger at the imposed slow versus the natural velocity (*F*(1,17) = 20.02, *p* < .001, *d* = 1.08, Fig. 3).

### Combined Influence of added mass and velocity

**Fig. 4:**
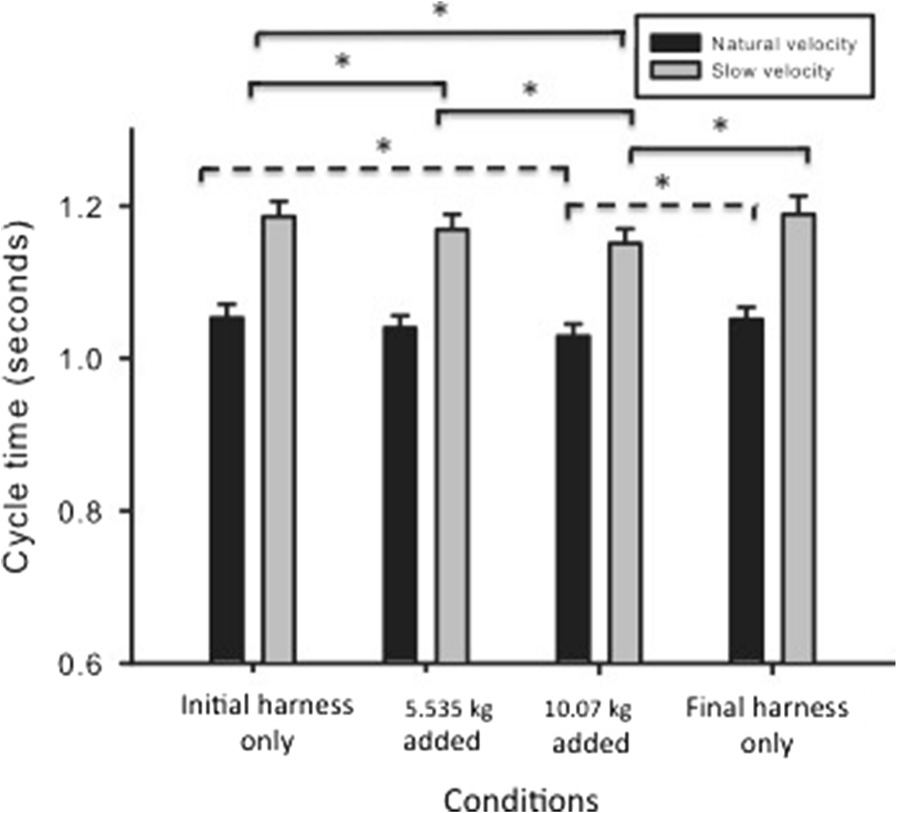
Spatio-temporal gait parameters by mass condition and walking velocity. Average cycle times are plotted for the initial harness only, 5.535 kg mass, 10.07 kg mass, and final harness only conditions at the natural (black bars) and slow (grey bars) velocities. Bars represents standard errors. * = *p* < .01 in which dashed lines are for the natural velocity and solid lines are for the slow velocity

We found an interaction between condition and velocity (*F*(3,51) = 6.98, *p* < .01, Fig. 4). At the natural velocity, the initial addition of the 5.535 kg mass resulted in no change in cycle time, and there were no differences from the 5.535 kg to the 10.07 kg added mass conditions. However, when wearing a 10.07 kg added mass at the natural velocity, participants decreased cycle time in comparison to the initial harness only condition (*p* < .01, *d* = 1.45). Once the anterior mass was removed, they increased cycle time during the final harness only condition at the natural velocity (*p* < .001, *d* = 1.33). In contrast, at the imposed slow walking velocity, cycle time decreased from the initial harness only condition to both the 5.535 kg (*p* < .001, *d* = 0.50) and 10.07 kg (*p* < .001, *d* = 1.50) added mass conditions, and from the 5.535 kg mass condition to the 10.07 kg added mass condition (*p* < .01, *d* = 1.00). Once the anterior mass was removed, they increased cycle time during the final harness only condition at the imposed slow velocity (*p* < .01, *d* = 1.50). No effects were found for the combined influence of velocity and mass on stride width, stride length, or double limb support time (*p* > .05). There were also no significant interaction effects for our hip, knee, or ankle kinematic variables (*p* > .05).

## Discussion

The purpose of this study was to examine the influence of added anterior mass on spatio-temporal and kinematic gait parameters when walking at natural and imposed slow velocities. To safely test the effects of anterior mass on gait and to avoid the effects of hormonal changes associated with pregnancy, we created a paradigm to use with healthy, non-pregnant women. The findings showed that adding mass decreased cycle time, stride length, and peak hip extension angles. Removing mass decreased stride width. Imposing a slow walking velocity resulted in an increase in cycle time and double limb support time and a decrease in stride length, peak hip extension angle and peak plantarflexion angle. Both added mass and an imposed slow walking velocity influenced cycle time; participants’ cycle time decreased with the addition of mass and increased once the additional mass was removed. Below, we discuss the impact of added mass and imposed slow walking velocity on the risk of falls.

The addition of anterior mass influenced participants’ gait. The alterations in gait that occur during pregnancy increase the risk of falling for pregnant women [12] even though these modifications may seem to increase stability [11]. Our findings confirm results from previous studies on how an increase in mass during pregnancy influences gait [4–6]. Other studies have shown how added mass influences adult gait, but they either did not add mass anteriorly [21] or focused on other aspects of gait such as toe clearance during obstacle crossing [22]. Although our participants were healthy women who were not pregnant, added anterior mass caused them to demonstrate spatio-temporal and kinematic gait patterns similar to those of pregnant women. We found that removing mass affected subjects’ stride width, but walking velocity did not influence stride width. Specifically, when walking with the 10.07 kg mass, subjects had larger stride widths compared to stride width during the final harness only condition. Our subjects only altered stride width when the heaviest mass was removed. Pregnant women demonstrate increases in step width linearly over the course of pregnancy [5]. The change in step width only occurring once the weight was removed could have been due either to participants adapting to wearing the harness or the fact that there was a sudden change. Our results may be highlighting non-pregnant women’s ability to compensate for a minor addition in mass (e.g., 5.535 kg) via spatio-temporal measures (e.g., decreasing cycle time). Larger additions in mass (e.g., 10.07 kg) may be required for non-pregnant women to demonstrate characteristic patterns in stride width similar to pregnant women. Still, it is notable that the largest mass condition in our study was enough to elicit gait adaptations similar to what is observed during pregnancy.

Our findings show that imposing a slow walking velocity led to higher cycle times, shorter stride lengths, longer double limb support times, smaller peak hip extension angles, and smaller peak plantarflexion angles. The 25 % imposed reduction in walking velocity is greater than the reported reductions in velocity with pregnancy that range from 5.6 % [8] to 15.6 % [23], but is within the range of changes that have been observed [6]. Imposed slower walking velocities are associated with higher fall risks unless paired with shorter stride lengths [24]. Walking at imposed slower velocities without concomitant decreases in stride length may increase balance constraints and increase the challenge of timing steps appropriately to avoid falling. Timing steps inappropriately leads to difficulty in recovering from a loss of balance [25]. With older adults, interventions aimed at timing steps to avoid falls [26] such as repetitive step training with prompts [27] have improved balance. For pregnant women with a history of falls, testing similar interventions to ensure that imposed slower velocities are paired with shorter stride lengths may be warranted based on our results.

Together, added anterior mass and the imposition of a slow walking velocity altered participants’ spatio-temporal gait parameters. At their natural walking velocity, participants’ gait modifications occurred in response to the addition and removal of the heaviest mass; they decreased cycle time in the 10.07 kg added mass condition and increased cycle time once the anterior mass was removed. Similar to walking at the natural velocity, when walking at the imposed slow velocity, participants increased cycle time when the anterior mass was removed. However, the combination of added mass and the imposed slow velocity caused participants to decrease cycle time during both added mass conditions. We did not find differences with kinematics possibly because our healthy subjects’ spatio-temporal parameters were most affected by the combined influence of mass and velocity.

### Limitations

One limitation includes not testing pregnant women, particularly those with a fear of falls. Pregnant women’s gradual increase in mass over 9 months allows them to adapt to biomechanical constraints [10]. In addition, we acknowledge that fear of falling is a main influence on adaptive changes in gait. Gait kinematics in pregnant women may be also affected by psychological factors that determine gait safety, which is related to both, the altered perception of their own movements, combined with increased pain and fear of balance loss [12, 28]. Fear of balance loss forces women to walk at a slower velocity, which can be achieved by shortening the step or decreasing gait cadence [5]. However, we aimed to first find a safe method for examining influences of added mass on gait without putting pregnant women at risk. Additionally, the use of non-pregnant women allows us to focus on acute spatio-temporal and kinematic changes independent of hormonal factors such as elasticity of the ligaments during pregnancy. We also did not assess the long-term effects of added mass on gait, which could be ameliorated by testing pregnant women longitudinally. Last, this study focused on treadmill walking and did not include the hormonal changes that occur during pregnancy. This study is the first step toward understanding how added mass and imposed slow walking velocities influence gait. Future studies need to be done to investigate the long-term effects of added mass (e.g., walking for an extended period of time with added mass). Even though perceptions of walking overground compared to walking on the treadmill differ, studies have demonstrated minimal measured kinematic differences when controlled for speed [29]. However, it may be useful to examine the effects added mass during overground walking compared to treadmill walking.

## Conclusions

Both added mass and imposed slow walking velocity resulted in gait modifications. These results suggest that the ways in which adults alter their gait may be commensurate with the magnitude of the additional mass when they walk at imposed slow versus natural velocities. This study presents a method for understanding how increased mass and imposed speed might affect gait independent of other effects related to pregnancy. Examining how added body mass and speed influence gait is one step in better understanding how women adapt to walking under different conditions.

### Availability of data and materials

The datasets supporting the conclusions of this article are included within the article. Any further information can be acquired from the authors upon request.

## Abbreviations

RM ANOVA: repeated measures analysis of variance
Kg: kilograms
Lbs: pounds
m/s: meters per second
m: meters
SD: standard deviation
